# Critical barriers to the large scale commercialization of silicon-containing batteries

**DOI:** 10.1039/d0na00589d

**Published:** 2020-08-26

**Authors:** Joseph Schwan, Giorgio Nava, Lorenzo Mangolini

**Affiliations:** Mechanical Engineering Department, University of California Riverside Riverside CA 92521 USA lmangolini@engr.ucr.edu; Materials Science and Engineering Program, University of California Riverside Riverside CA 92521 USA

## Abstract

Silicon has received a considerable amount of attention in the last few years because of its large lithiation capacity. Its widespread utilization in real-life lithium-ion batteries has so far been prevented by the plethora of challenges presented by this material. This review discusses the most promising technologies that have been put forward to address these issues. While silicon is now much closer to being compatible with commercial-grade storage devices, some critical barriers still deserve further attention. Most importantly, device performance is strongly dependent on particle size and size distribution, with these parameters strongly controlled by the particle synthesis technique. Moreover, the nanoparticle synthesis technique ultimately controls the material manufacturing cost and compatibility with large-scale utilization. These issues are discussed in detail, and recommendations to the community are provided.

## Abstract 2

The progress towards the integration of silicon into commercial batteries is critically assessed. Improving the quality of the silicon nanostructures is identified as one of the key areas requiring further attention.

## Graphical abstract

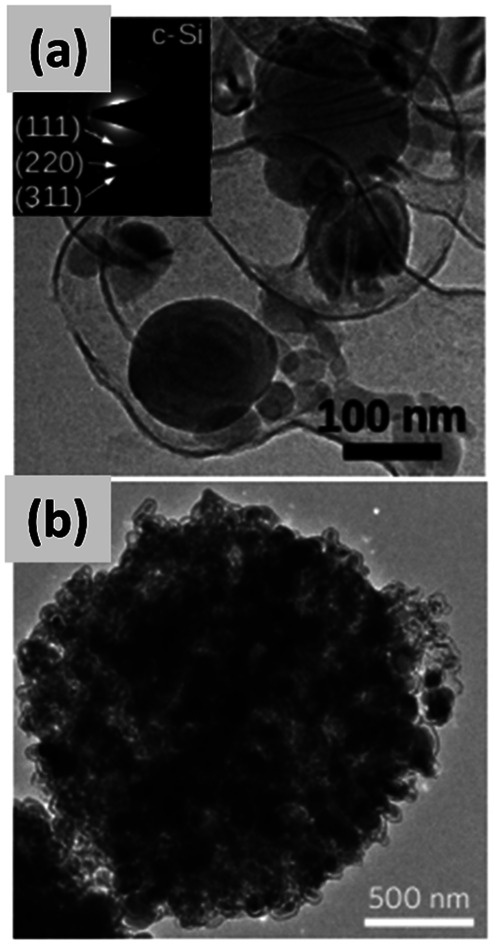

## 1. Introduction

The successful integration of silicon into lithium-ion batteries has been sought after for almost two decades,^1–5^ with considerable efforts from academic research groups, national labs and industrial entities from all over the world. The interest in silicon as new battery material is motivated by its high energy density, both on a volumetric and gravimetric basis.^6^ These are considerably higher than the case of graphitic carbon, the current industry standard. The lithium-ion battery market, which was approximately 343 billion dollars in 2018, is expected to reach almost 900 billion dollars by 2025, with a projected compound annual growth rate (CGAR) of roughly 15%.^7^ This growth, while rapid, is limited by the storage capacity of current materials, with graphite still being the dominant technology for anodes. There is an exceptional need for new material chemistries that increase energy density, driven by fields ranging from renewable energy to aerospace. Despite its high lithiation capacity, silicon presents unfavorable characteristics; most notable is the fact that it undergoes significant volume changes during lithiation and delithiation.^3,4^ While this issue can be alleviated by reducing silicon's dimensionality to nanoscale,^5^ this approach introduces additional problems. The high surface area of nanoscale materials tend to decrease first cycle coulombic efficiency due to the irreversible consumption of lithium by the solid electrolyte interphase (SEI).^6^ Volume changes, still occurring even at nanoscale, further destabilize the solid electrolyte interphase (SEI) with detrimental effects on cycling stability.^6,8,9^ Ensuring good electrical contact over many charge–discharge cycles when the active component varies in volume also presents a challenge.^10^ These issues will be discussed in details in this manuscript. The combination of these obstacles has proven a difficult roadblock to enabling the use of silicon as anode material. Despite this, significant progress has been made towards alleviating these issues. Silicon is now closer to mass-scale, real-life utilization in batteries. Aside from the clear socio-economic need for silicon-containing batteries, the fundamental scientific problems posed by silicon in this application space has piqued the interest of the community at large. This community has risen to the challenge by designing and synthesizing novel nanostructures,^11–13^ implementing advanced *in situ* characterization tools,^14^ experimenting with novel binder^15,16^ and electrolyte chemistries,^17^ and by applying refined computation tools to this problem.^18^

For all these reasons, the literature in the area of silicon for batteries is large and still rapidly growing, and thus non-trivial to navigate. The first goal of this review is to provide a useful guide to researchers by summarizing the most significant and recent advances in this area. The second goal is to critically assess which areas require further attention by the community. For example, one important issue that has been overlooked by the community, and that will be discussed at length, is the quality of the silicon nanomaterials used in experiments. Even subtle changes in seemingly minor factors such as particle size distribution greatly affect battery performance. Moreover, there is also a significant number of techniques for silicon nanoparticle synthesis, with consequences on their cost and ultimately on compatibility with commercial large-scale applications.

We stress that this document specifically focuses on silicon in the form of nanoparticles or nanopowders. Silicon nanowires^19–23^ and thin films^3,24–27^ have also been investigated at length, but the powder format is easier to manufacture and easier to integrate into current industrial schemes, which typically involve the development of slurries that can be coated onto current collectors in a roll-to-roll manner. Seamless integration into existing industrial production schemes and methods is considered as a fundamental requirement for the adoption of new materials by the battery manufacturers.

This review is organized into two primary sections: Section 2, which focuses on the most promising and recent advances in the design and production of silicon-containing anodes, with each subsection highlighting the technologies that have been proposed to address the specific weaknesses of silicon as anode materials, *i.e.* swelling upon lithiation, poor electrical conductivity, need for specialty binders and electrolyte chemistries; Section 3, which provides a comprehensive discussion of the synthesis of silicon nanoparticles. Section 4 highlights the areas that require further development and provides recommendations to the community.

## 2. State-of-the-art in silicon-containing anodes

Despite its potential, successfully incorporating silicon into anodes for lithium-ion batteries will require a multi-prong approach that addresses many of the issues posed by this material. A large number of proposals have been advanced in the literature with respect of managing the problematic aspects of silicon. Each subsection focuses on the most promising approaches in each area.

### 2.1 Managing volume expansion

Early work on the lithiation of silicon clearly indicated that swelling upon lithiation is a major challenge.^3,4^ Pulverization of the active material and loss of electrical contact results in irreversible losses and high fade rate during cycling, making silicon in its bulk form unusable for any practical application. Liu *et al.*^28^ conclusively showed *via* detailed *in situ* TEM characterization that sufficiently small silicon particles do not fracture upon lithiation. The authors found that the critical size for this transition is ∼150 nm.^28^ This important result offers a practical approach towards stabilizing silicon, *i.e.* reduction to nanoscale. At the same time, it introduces additional issues that need to be addressed to achieve commercial-grade stability. Realizing a silicon-dominant electrode with reasonable areal capacity (3 mA h cm^−2^ or higher), high first cycle coulombic efficiency (>90%) and high stability (>80% capacity retention over hundreds of cycles) implies manufacturing a thick coating of silicon nanoparticles with high electrical conductivity to prevent the formation of electrical “dead zones”. This requires a binder that can withstand the large volume changes without preventing charge transport, and an electrolyte chemistry that minimizes the degradation induced by the unstable SEI layer. This is no easy task.

**Fig. 1:**
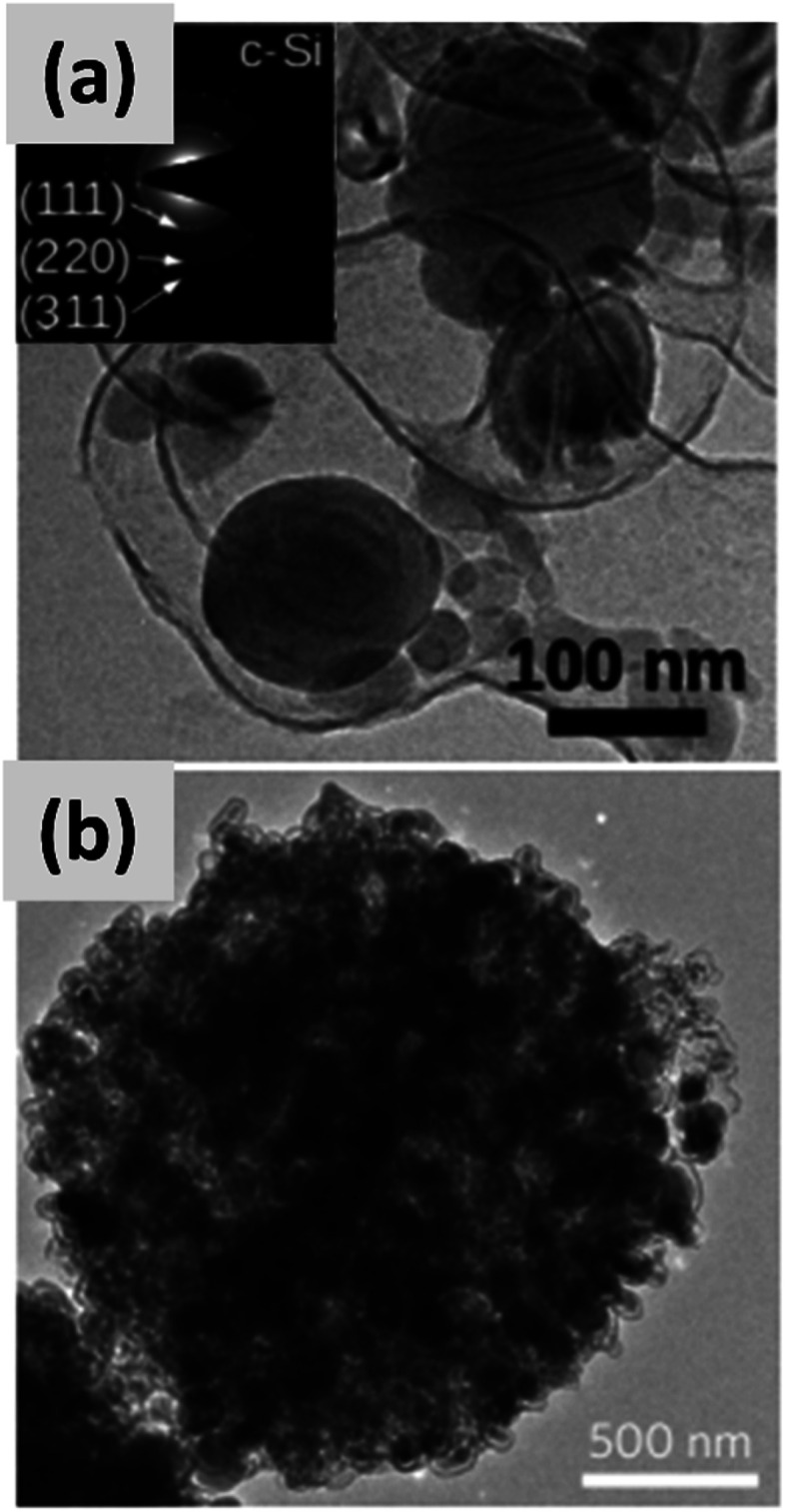
(a) TEM of a single yolk–shell silicon–carbon nanoparticles. Reproduced with permission from ref. 11. (b) TEM of a ∼1 μm particle formed *via* the controlled agglomeration of yolk–shell silicon–carbon nanoparticles. Reproduced with permission from ref. 29.

**Fig. 2:**
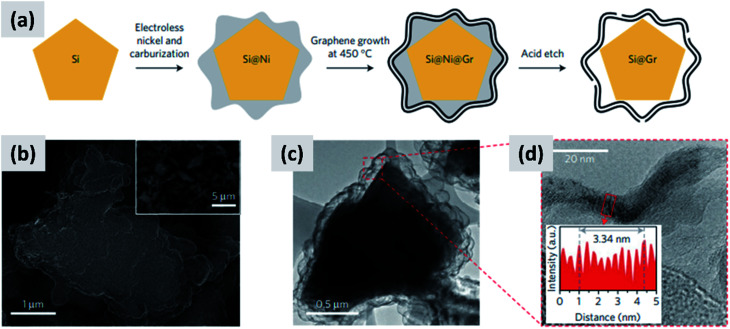
(a) Process schematic for the synthesis of yolk–shell silicon–graphene nanoparticles. (b) and (c) are SEM and TEM images of the graphene-encapsulated silicon particles, respectively. (d) Higher magnification TEM micrograph of the graphene shell, confirming its layered structure. Reproduced with permission from ref. 30.

The yolk–shell structure appears to be a promising strategy to meeting these requirements. This design has been championed by many groups with many advancements being attributable to the Cui group.^11,29,30^ The design involves the encapsulation of silicon particles into a shell with a carefully engineered void or buffer space to accommodate for the volume changes at the single particle level. By giving room for each particle to swell upon lithiation, it is possible to realize a coating that is structurally and electrically stable, effectively removing all of the issues listed above. The drawback of this approach is the fact that it requires a very careful design and a complex synthesis protocol. In a first report, Liu *et al.*^11^ discuss the production of such structures using commercially available silicon particles around 100 nm in size which are then coated with a silica shell by liquid-phase polymerization using tetraethyl orthosilicate (TEOS) as precursor. A carbon coating is added by polymerizing polydopamine onto the silicon–silica core–shell, followed by carbonization at high temperature. The silica layer is then removed by a hydrofluoric acid treatment. The resulting structure, show in Fig. 1a, contains roughly 70% of silicon by weight, as determined by thermogravimetric analysis (TGA), and shows a capacity ∼ 1500 mA h g^−1^ with excellent stability even after 1000 cycles when tested in a half cell. Noteworthy is the high coulombic efficiency (CE) reached by the structure, exceeding 99.8% and confirming the formation of a stable SEI layer. A further improvement on this concept is discussed by Liu *et al.*^29^ The authors use the same yolk–shell particles just discussed, but then apply a micro-emulsion technique to assemble the particles into multi-particle aggregates (a representative TEM is shown in Fig. 1b). This results in a decrease in the effective surface area of the material and in additional improvements in stability. Three noteworthy observations are reported in this study. First, results for anodes with a weight loading as high as ∼3 mg cm^−2^ are presented. That loading corresponds to an areal capacity of ∼3 mA h cm^−2^, in line with commercial requirements. Second, the author discuss the use of calendering on their material. They report that calendering is compatible with the structure, achieving a volumetric capacity as high as ∼1200 mA h cm^−3^ which is roughly double that of graphite. Finally, the authors^29^ discuss the first cycle coulombic efficiency (CE) of their anodes. The find that an increase in the thickness of the outer carbon shell correlates with a decrease in first cycle CE. This strongly suggest that while the SEI is expected to be stable due to presence of a buffer space between the silicon core and the carbon layer, the amorphous carbon shell may irreversibly trap lithium during cycling. The quality of the carbon shell is an important parameter that affects the overall anode performance. Li *et al.*^30^ describe the realization of a high-quality multi-layer graphene shell as outer layer of the yolk–shell structure. The synthesis protocol (see Fig. 2a) involves multiple steps including the electrodeless plating of nickel on top of micron-sized silicon particles, the carburization of the core–shell structure to nucleate and grow the graphene layer, and multiple etching steps to remove the catalyst nickel layer and the oxide diffusion barrier present at the surface of the silicon powder. The presence of both a high-quality graphene shell and of a buffer space between the silicon particle and the shell, shown in Fig. 2b–d, translates into what is probably the best performance reported to date for a silicon-dominant anode. The authors report a first cycle CE of 92% and excellent stability at areal loadings approaching 3 mA h cm^−2^. Most importantly, the authors also present very encouraging data for a full cell (coin sized) using lithium cobalt oxide (LCO) as cathode, with stability comparable to that of a half cell over 100 cycles at C/2 charge–discharge rate. These reports^11,29,30^ unequivocally suggest that managing the volume expansion of silicon during lithiation greatly improves its stability. A potential drawback of yolk–shell structures is the complexity of their synthesis protocols, which involve the deposition of sacrificial layers and multiple deposition and etching steps. This raises questions about their compatibility with large-scale manufacturing requirement. A precise cost analysis of the synthesis scheme is beyond the scope of this review, and ultimately viability will only be verified by its successful commercialization.

**Fig. 3:**
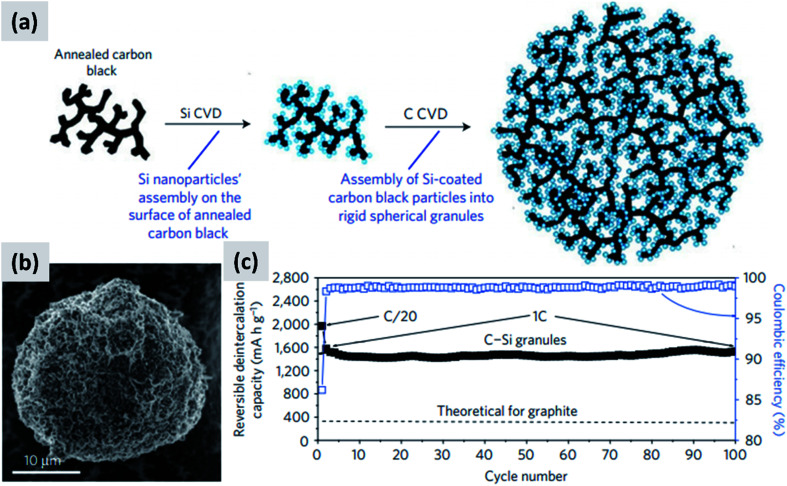
(a) Process schematic for the synthesis of silicon–carbon composite granules. (b) SEM image of the granules. (c) Delithiation capacity for a coin sized half-cell. Reproduced with permission from ref. 12.

Another approach that successfully manages the volume changes of silicon during cycling is the one described by Magasinski *et al.*^12^ This is based on the realization of a conductive carbon scaffold *via* the partial sintering of carbon black powder, followed by infilling with silicon using thermally-activated chemical vapor deposition (CVD). An additional carbon coating is then applied to realize micron-size agglomerates, although these are porous and have a large surface area (24 m^2^ g^−1^). The synthesis is summarized in Fig. 3a, with Fig. 3b showing a SEM image of the resulting particle. Despite the high surface area, this material has a first cycle CE of 85% and shows excellent stability over 100 cycles at 1C (Fig. 3c). The volumetric capacity of the assembly exceeds 1200 mA h cm^−3^, *i.e.* double that of graphite. The good performance of this structure is likely due to the electrical conductivity and structural stability of the carbon scaffold, and to the porosity of the structure which provides sufficient buffer space for silicon to expand. While full cell data are not presented in ref. 12, and the weight loading of the tested anode is not disclosed, the excellent half-cell data suggest that this approach would probably perform well in a full-cell. It is noteworthy that a very different synthesis approach is followed in this study, as compared to those proposed by the group of Cui^30^ for yolk–shell particles. Gas-phase chemical vapor deposition is used to grow silicon and carbon layers, using precursors such as silane (SiH_4_) and propylene (C_3_H_6_). Gas-phase processing techniques (1) can be precisely tuned by controlling time–temperature combination, (2) can achieve highly conformal coatings when operated in a surface reaction limited regime, and (3) avoid the use of solvents which are often difficult and expensive to dispose of. While gas-phase precursors for carbon are generally inexpensive and readily available, silane requires special handling due to its pyrophoric nature and is expensive (NREL reports a cost of $184 per kg for 6 N silane, corresponding to $210 per kg of silicon^31^). Therefore while it is possible to realize conformal silicon coatings even on highly porous materials by operating in a surface reaction limited regime (*i.e.* at low-to-moderate CVD temperature), this may prevent the full utilization of a costly precursor. The authors in ref. 12 do not discuss the silane utilization efficiency for their synthesis process.

### 2.2 Improving conductivity

Nominally undoped silicon has low electrical conductivity, significantly lower than that of graphitic carbon. The fact that it needs to be nano-sized to prevent pulverization exacerbates this issue. Silicon-dominant anodes are typically composed of micron-thick layers of nanoparticles, greatly reducing charge transport through the network. A broad range of strategies have been tested to overcome this obstacle.

**Fig. 4:**
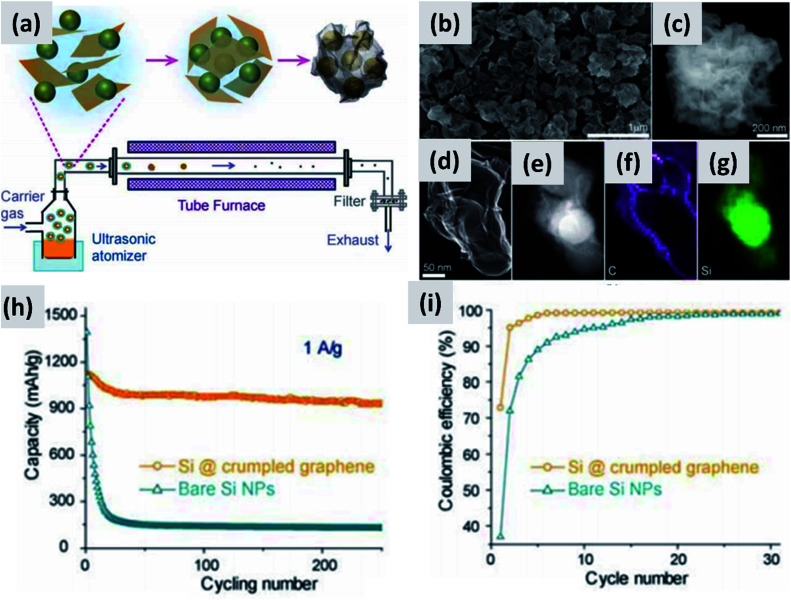
(a) Schematic of a spray-drying process for the encapsulation of silicon particles in crumpled graphene. (b and c) SEM images of the assembled particles. (d) TEM image of single silicon particle encapsulated in graphene. (e) Z-contrast showing the presence of a silicon core. (f and g) Elemental maps for carbon and silicon respectively. (h) and (i) are capacity and coulombic efficiency for this material, compared to the control sample composed of bare silicon nanoparticles. Reproduced with permission from ref. 39.

The increase in interest in silicon for batteries has overlapped with the rise of graphene as a novel nanomaterial with outstanding electrical and thermal transport properties.^32–36^ It was therefore natural for several groups to investigate the combination of graphene and silicon as anode structure.^13,37–41^ One of the early reports on this approach is from Lee *et al.*^37^ The authors describe the fabrication of a graphene-based paper that effectively incorporates silicon particles. This utilizes graphene oxide (GO) as building block together with commercial ∼30 nm silicon particles as the dominant active material. The two components are mixed in water together with a binder, cast to form a coating, and annealed in forming gas to reduce the graphene oxide to conductive graphene. The authors use oxide-free silicon particles as feedstock, but they let the particles oxidize to improve their dispersion in water. The performance of half-cell based on this structure is good, with high initial capacity (∼2500 mA h g^−1^) although the capacity drops by almost 30% after 300 cycles and the initial CE is not discussed. Moreover, the coating is relatively thin (5 μm), suggesting that charge and lithium transport through the assembly may require further improvement. Very similar performance is also reported by Zhou *et al.*^38^ who use freeze-drying to create a good dispersion of ∼300 nm silicon particles into graphene oxide sheets. A thermally-active reduction step is again needed to reduce GO to graphene and enable functionality. Another interesting example is provided by Luo *et al.*^39^ who first create a mixture of graphene oxide flakes and silicon particles in water, and then use aerosol spray pyrolysis to encase the particles into micron-size crumpled graphene structures, as shown in Fig. 4. This result is interesting because spray drying is a widely utilized industrial process therefore compatible with large scale manufacturing requirements, and the final result is a powder which can then be applied onto copper foil using standard coating technique. The performance of the structure is good, with >80% retention over 200 cycles, although the first cycle CE is around 70% and the weight loading is somewhat low (0.2 mg cm^−2^). In the work of Greco *et al.*,^40^ the authors demonstrate that graphene flakes produced by liquid phase exfoliation of graphite improve the stability of silicon-based anodes compared to amorphous carbon and graphene oxide. Overall these results suggest that graphene greatly improves cycling compared to silicon anodes, although its stability is not compatible yet with commercial applications. One issue is the inherently large surface area of graphene, which is likely conducive to poor first cycle CE.^35,42,43^ Another issue is the nature of the contact between the current carrying component (graphene) and the lithium-storage component (silicon). Evanoff *et al.*^13^ use silane as CVD precursor and applied a silicon coating directly onto multi-layer graphene flakes. The resulting structure shows a ∼60% first cycle CE, but the stability over 150 cycle was clearly improved compared to the case in which silicon particles and graphene flakes are simply mixed. Noteworthy is the manuscript from Ren *et al.*,^41^ which also describes a technique for the direct deposition of silicon onto graphene flakes *via* CVD, but using trichlorosilane (SiHCl_3_) instead of silane (SiH_4_) as precursor. Trichlorosilane presents advantages compared to silane in terms of cost (it is the main precursor for the production of polysilicon, which is priced at $15 per kg (ref. 44)) and ease of handling, since it is a liquid at room temperature compared to a pyrophoric gas. The performance reported by Ren *et al.*^41^ is indeed quite similar to that reported by Evanoff *et al.*^13^ The group also experimented with applying a thin alumina coating onto their structure *via* atomic layer deposition (ALD), with a small but significant improvement in capacity and stability. Finally, a survey of this subfield has revealed that the term graphene is loosely applied to single layer, multilayer and thick flakes, making a direct comparison between these studies quite difficult.

Similarly to the case of graphene, combining silicon with carbon nanotubes has also attracted the attention of several groups.^45–49^ One crucial issue in this heterostructures is the binding of silicon to the nanotubes. Martin *et al.*^45^ discuss a chemical route to bind carbon nanotubes and silicon nanoparticles which is based on the functionalization of the nanotubes with phenyldiamine. Simple mixing of water-soluble silicon nanoparticles with the functionalized nanotubes allows the formation of a covalent bond between the two components. The appeal of this approach lies in its simplicity, in the fact that it uses readily available components, and in the use of water as solvent. On the other hand, the resulting anode suffers from larger than expected capacity fade. Wang *et al.*^46^ and Evanoff *et al.*^47^ use CVD to first grow carbon nanotubes directly onto the copper current collector, and then use CVD of silane to coat the nanotubes with a silicon layer. The resulting performance is good, with first cycle CE exceeding 80% in both cases and promising stability over few hundreds of cycles. One important difference between these two reports is the fact that the anode is realized using vertically-aligned nanotubes grown directly from the copper current collector for the contribution from Evanoff *et al.*^47^ Wang *et al.*^46^ instead mechanically remove the nanotube–silicon heterostructures and then develop a slurry which is applied onto a current collector. These two techniques yield similar results in performance, although neither of these two papers discuss the weight loading and the volumetric energy density of their anodes. Carbon nanotubes have also been used to realize free-standing electrodes, *i.e.* anodes that do not require a copper foil as current collector. This concept is demonstrated by Evanoff *et al.*^48^ and Cui *et al.*^49^ who use nanotubes to realize a fabric with excellent mechanical properties and infilled with silicon using CVD. Evanoff *et al.*^48^ report a capacity of 500 mA h per gram with excellent stability and ∼90% first cycle CE. The authors report a volumetric capacity of 640 mA h cm^−3^. Most importantly, they suggest that the actual capacity of graphite-based anodes is around 200 mA h per gram when accounting for the weight of the copper current collector. Therefore removing it provides an immediate improvement in energy density. The anode realized by Cui *et al.*^49^ is similar, although the capacity is even higher (∼2000 mA h per gram) because of the growth of a quite thick silicon layer around the carbon nanotubes, to the point that the structure resembles more a fiber-reinforced silicon film rather than a fabric. They report a ∼20% improvement in energy density at the whole battery level when employing this structure.

Another approach to improving the charge transport properties of silicon-containing anodes is to carbon coat the silicon particles. This strategy has received considerable attention. The large variety in silicon powders and carbon coating techniques make this category particularly difficult to review, and it is challenging to compare results reported by different groups. Still, some broad guidelines can be derived by critically analyzing the literature on this sub-topic. First of all, several early reports on the coating of silicon particles with carbon use a polymer precursor to create a shell structure, followed by annealing at high temperature to carbonize the polymeric shell into an amorphous/glassy carbon structure.^50–57^ Several different polymers can be used to that end. Hu *et al.*^50^ use an hydrothermal treatment to first grow a carbon layer around 30–50 nm silicon particles using glucose as precursor. Annealing in an inert atmosphere at 750 °C for 4 hours leads to silicon/carbon core/shell particles. Raman characterization shows broad D and G peaks with a high D/G ratio, suggesting that the carbon shell is partially graphitized. Coating of this material using carbon black and PVDF as binder leads to stable capacity retention, although the first cycle CE is quite low (∼60%). It should be noted that the authors stress that the addition of vinylene carbonate (VC) is crucial for improving the stability of the device.^50^ Also, the synthesis technique inevitably leads to partial oxidation of the silicon particles. Disproportionation of the silicon oxide into silicon and lithium oxide during the first cycle leads to an irreversible loss of lithium, lowering first cycle CE. Gao *et al.*^51^ use an ultrasonic-assisted polymerization technique to coat 80–120 nm silicon particles with poly(cyclotriphosphazene-4,4′-sulfonyldiphenol) (PZS). The technique allows maintaining the silicon particle separated, improving the quality and the uniformity of the coating. Pyrolysis is then performed at 900 °C in argon. The resulting anode has ∼1200 mA h per gram capacity with a ∼70% first cycle CE. The authors use nitrogen adsorption–desorption and find that the carbon shell is actually porous with an average pore size around 0.7 nm. In a series of contributions, Ng *et al.*^52–54^ use spray pyrolysis to overcoat 100 nm silicon particles with amorphous carbon using citric acid as precursor. The particles and citric acid are dispersed in ethanol. The solution is nebulized and flown through a furnace at 400 °C. The authors demonstrate good control of the silicon-to-carbon ratio, which is adjusted by varying the pyrolysis temperature in the 300–600 °C range. Unfortunately, the use of higher temperature is not discussed. The amorphous nature of the carbon shell is determined by the lack of a graphite peak in the XRD spectrum, although no Raman data is presented. No further pyrolysis or heat treatment is applied to the material, which shows reasonable performance with ∼1500 mA h per gram capacity and a 70% first cycle CE. Most importantly, the authors perform electrochemical impedance spectroscopy (EIS) indicating that anodes composed of carbon-coated silicon particles have a significantly reduced impedance compared to the case of bare silicon particles, confirming the improved conductivity of the nanoparticle network when the carbon shell is applied. Lee *et al.*^55^ and Jung *et al.*^56^ discuss the formation of a carbon shell around silicon particles by using a resorcinol-formaldehyde resin. In both of these contributions, the silicon particles are first surface modified to improve their dispersion in the resin, followed by curing over extended period of time and pyrolysis at high temperature. The performance in these two reports is similar, with reasonable stability but somewhat low first cycle CE (<70%). Another variation on the use of polymers for growth of the carbon shell is presented by Guo *et al.*,^57^ who first functionalize 20–30 nm silicon particles with hydrophilic and vinyl groups to disperse them in water and use them as seeds to drive the polymerization of acrylonitrile. The resulting core–shell silicon–polyacrylonitrile (PAN) structure is carbonized at 900 °C for 1 hour. The resulting structure shows a high D/G Raman peak ratio, suggesting that the carbon shell has high quality. The cycle stability is quite good, although the first cycle CE is low (∼20%), again attributed to the significant oxidation of the silicon particles during the polymerization procedure.

**Fig. 5:**
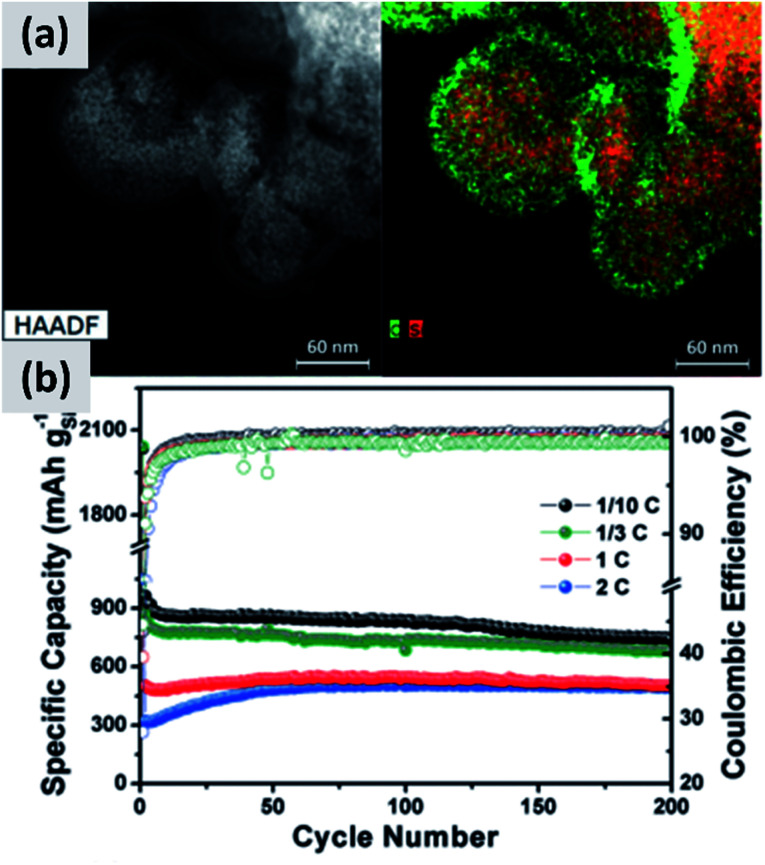
(a) TEM of small (<10 nm) silicon particles embedded in a polystyrene-derived carbon shell, with the corresponding elemental mappings for carbon and silicon. (b) Cycling data and coulombic efficiency for this structure, at varying C rates. Reproduced with permission from ref. 59.

The use of polymeric precursors has also been explored as a way to bind together aggregates of silicon particles, with the goal of realizing micron-sized structures with very fine crystalline domains. This structure could then be resilient to pulverization upon lithiation while at the same time have low surface area, which would be beneficial from a coulombic efficiency point of view. Zhong *et al.*^58^ use very small silicon particles (∼5 nm) and disperse them in a poly-vinylpyrrolidone (PVP) matrix. After annealing in argon at 700 °C, the polymer forms large micron-sized beads. The resulting anode shows excellent stability, with *ex situ* characterization confirming that the large agglomerate do not crack even after several charge–discharge cycles, although the first cycle CE (∼60%) is not compatible with commercial applications. A similar approach is presented by Su *et al.*^59^ In this study, small silicon nanoparticles (∼5 nm) are first surface-modified with alkyl groups to make a colloidal dispersion in non-polar solvents. A micro-emulsion technique is then followed to uniformly disperse the silicon particles into a resorcinol-formaldehyde matrix. After pyrolysis, the silicon particles are uniformly dispersed in hollow carbon structures, as shown in Fig. 5. Anodes based on this material shows excellent stability with 900 mA h g^−1^ capacity and very little decay over 200 cycles, although the first cycle CE is 43%. Lu *et al.*^60^ discuss yet another variation to this approach which is based on coating micron-size silicon monoxide (SiO) particles with a resorcinol-formaldehyde (RF) resin. Interestingly, the pyrolysis step converts the resin into a carbon layer and at the same time induces the disproportionation of the sub-oxide into silicon and silicon dioxide. Subsequent hydrofluoric acid treatment (HF) allows removing the oxide, realizing a core–shell silicon–carbon structure in which a void space is left in the core to accommodate for the volume expansion upon cycling. The structure shows excellent stability over 1000 cycles at C/4 rate, with a ∼1500 mA h per gram capacity and a ∼70% first cycle CE. The mass loading for this report is 0.6 mg cm^−2^, and the volumetric capacity at ∼1000 mA h cm^−3^ largely exceeds that of graphite.

Based on these contributions, it can be concluded that the presence of a carbon coating around the silicon particle is beneficial not only with respect of charge transport but also cycling stability. This is consistent with theoretical predictions suggesting that the carbon shell can effectively limit or prevent the pulverization of the silicon core upon lithiation.^18^ One common issue in these reports is the low first cycle coulombic efficiency. This is a crucial parameter which has to be as high as possible (ideally >90%) for successful integration with realistic cathode chemistries. Testing with lithium metal as counter-electrode (*i.e.* half-cell) is forgiving since the large reservoir of lithium is not present in real-life batteries. Many of the techniques described above use polymerization in water, sometimes following hydrothermal procedures, so that partial oxidation of the silicon core is inevitable. Therefore the presence of an oxide likely contributes to the low first cycle CE. In fact, Lu *et al.*^60^ report a reasonable first cycle CE only after treating their material with HF to remove the oxide from the structure.

Another issue that deserves further attention relates to the quality of the carbon coating. Carbon can be produced with a broad range of structures (from amorphous to partially or fully graphitic) and properties depending on processing conditions.^61–63^ It is therefore relevant to study the literature on the use of carbon as lithium-storage material. Amorphous or disordered carbon has received significant attention as lithium storage material. Endo *et al.*^64^ anneal poly-*p*-phenylene (P*P*P) carbon and report a capacity of 700 mA h per gram for polymer treated at 700 °C, with capacity decreasing for increasing annealing temperature. They correlate the increase in capacity, compared to graphite, with the small crystalline domain size obtained for the samples pyrolyzed at low temperatures, therefore hypothesizing that lithium can be stored at the boundaries of the crystalline domain. Tokumitsu *et al.*^65^ observe a similar behavior in carbon materials obtained *via* heat-treatment of condensed polynuclear aromatic carbon. They attribute the increase in capacity (as high as 1200 mA h g^−1^) to the presence of sub-nanometer voids formed when annealing at low temperatures (as low as 600 °C). Dahn *et al.*^66^ correlate the increase in lithium capacity with high H/C atomic ratio, as high as 0.4 for a ∼1000 mA h per gram value. The probability of observing single layers in the carbon structure, *i.e.* the lack of stacking or the increase in structural disorder, also correlates with an increase in lithiation capacity. Zheng *et al.*^67,68^ have discussed the performance of carbon materials as obtained *via* the pyrolysis of phenolic resins, petroleum pitch, polyvinylchloride, polyvinylidene, polyphenylene sulfide. Liu *et al.*^69^ investigated carbon produced from epoxy resins, and Buiel *et al.*^70^ focuses on sucrose as precursor. These studies confirm that an increase in disorder and hydrogen content in carbon correlates with an increase in capacity, but that the increase is inevitably accompanied by a fast decay over few charge–discharge cycle, and that the first cycle irreversibility can be very large. For instance, the carbon derived from phenolic resin discussed in ref. 67 has a relatively stable capacity of 550 mA h per gram when annealed at 1000 °C, but the first cycle capacity is 750 mA h per gram, implying a first cycle CE of only ∼70%. This value is even worse at lower annealing temperatures. It is interesting to point out that Xing and Dahn^71^ report an important decrease in irreversible losses for pyrolyzed carbon samples which are never exposed to air, leading to the hypothesis that adsorption of CO_2_, O_2_ and water from air is particularly detrimental in these materials due to their high effective surface area. These species oxidize lithium during the first battery cycle in an irreversible manner. Buiel *et al.*^70^ expose the carbonaceous sample to ethylene during pyrolysis, leading to a decrease in surface area as measured by the BET method (from ∼250 to ∼20 m^2^ per gram) and to a significant decrease in irreversible capacity losses, consistent with the previous observation. Based on these reports, it can be concluded that the low first cycle CE which is often observed in silicon–carbon composite anodes is at least partially due to the carbonaceous component. This issue can be controlled by annealing at higher temperature (1000 °C or above), although other problems may rise such as the formation of lithiation-inactive silicon carbide,^72^ which is likely to be kinetically favored in nanosized systems due to large interface area.

**Fig. 6:**
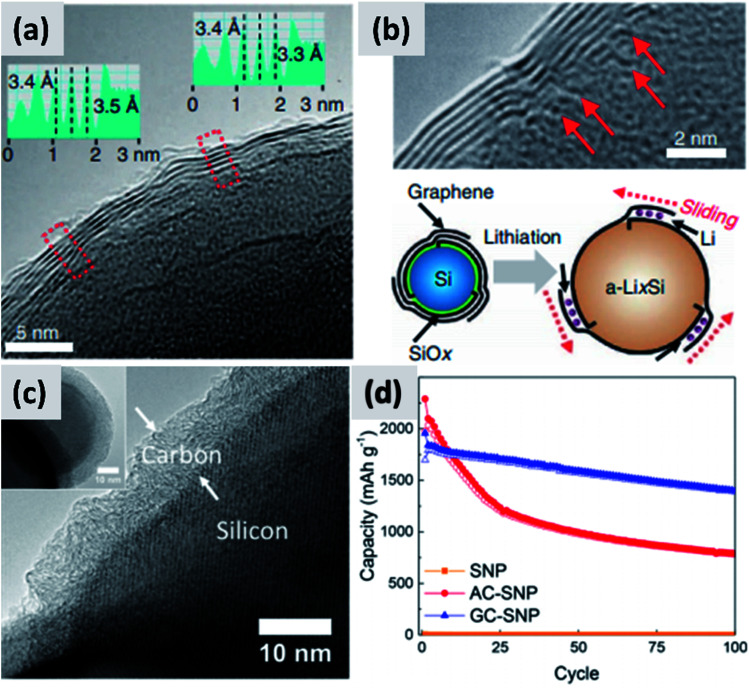
(a) TEM of a multi-layer graphene shell grown on silicon *via* CVD, using CO_2_ as mild oxidizing agent. (b) Higher magnification TEM for (a), with schematic of the proposed stabilization mechanism. Reproduced with permission from ref. 73. (c) TEM of a graphitized carbon shell grown on silicon *via* CVD using a carefully optimized heating procedure. (d) Performance comparison between particles with amorphous (AC-SNP) and graphitized (GC-SNP) carbon shells. Reproduced with permission from ref. 75.

An alternative and promising approach is the use of vapor phase CVD processes for the growth of high-quality carbon on silicon nanostructures. By carefully controlling the combination of growth time and temperature, it is possible to realize conformal coatings on nanoparticles with arbitrary thickness and a high degree of graphitization. Particularly interesting are the reports from Son *et al.*^73,74^ describing the addition of carbon dioxide (CO_2_) to methane (CH_4_) when growing a carbon shell at 1000 °C around commercial ∼100 nm silicon particles. CO_2_ acts as a mild oxidizing agent which selectively removes defects during the growth of the carbon layer. The result is a highly uniform and conformal multi-layer graphene coating on silicon particles, as demonstrated by TEM (Fig. 6a and b) and by very conclusive Raman characterization showing narrow D and G peaks and clear signature of the 2D peak. Control experiments performed using hydrogen in place of methane also lead to highly graphitized coatings, although not as conformal as in the case of CO_2_. Moreover, CO_2_ is found to be instrumental at preventing the formation of interfacial SiC, which would be detrimental from a capacity and lithium transport point of view. The resulting anodes show >80% first cycle CE and ∼90% capacity retention at 100 cycle when the areal capacity is 3 mA h cm^−2^. The volumetric energy density is excellent at almost 3000 mA h cm^−3^. The authors attribute the high stability to the capability of the graphene layers to slide against each other and accommodate for the volume chance of the underlying silicon particles. Most importantly, the authors discuss in details the integration of the material in a full cell with LiCoO_2_ (LCO) as cathode. While the capacity decay is faster than in the case of the half-cell, as expected, the same areal capacity is provided by an anode which has a thickness of ∼100 μm, compared to the ∼170 μm thick graphite coating. The overall battery energy density increases from 550 W h L^−1^ to 972 W h L^−1^. Nava *et al.*^75^ have investigated the use of CVD of carbon to stabilize ∼100 nm silicon particles. By carefully controlling the heating profile of the material during CVD of acetylene, it is possible to grow uniform carbon shell with control not only over its thickness but also over its structure (Fig. 6c), all while avoiding the formation of a silicon carbide interfacial layer. This allows providing a direct comparison between amorphous and graphitic shell structures, shown in Fig. 6d. Anodes prepared with the graphitic shell have ∼86% first cycle CE with ∼80% capacity loss over 100 cycles at 0.1C rate, demonstrating a significant improvement in stability compared to the case of the amorphous carbon shell. These results clearly confirm that graphitic carbon is highly preferable for two reasons: first, graphitic carbon has significantly better conductivity, improving charge transport and most likely preventing the formation of electrically “dead zones” in the anode assembly; second, the graphitic shell can better accommodate the swelling of the silicon core, thereby maintaining good structural stability and electrical conductivity over repeated charge–discharge cycles, as confirmed by EIS measurements. The improved mechanical resilience of graphitic carbon as opposed to amorphous carbon has been confirmed by *in situ* TEM by Li *et al.*^30^ This is an aspect that deserves further attention from the community, as it is highly consequential for the functionality and successful development of silicon-based anodes.

It is also important to mention that while the vast majority of the community has focused on using carbon to improve conductivity and stability of silicon-based anodes, other materials have been evaluated for the same purpose. Chevrier *et al.*^76^ discuss the development of a silicon-containing micron-sized particles which is referred to as composed of an active-inactive alloy. The structure shows significant improved stability compared to commercially available silicon particles. Zhong *et al.*^77^ decorate the surface of silicon particles with tin nanocrystals obtained by the reduction of a readily available tin salt. The authors observe a reduction in impedance and an improvement in stability compared to the silicon-only case. Finally, the use of 2D materials other than graphene has also been considered. Zhang *et al.*^78^ propose the use of MXenes for the preparation of a silicon-containing, highly conductive and mechanically stable scaffold, resulting in anodes with outstanding areal capacity, as high as 20 mA h cm^−2^.

Overall, the scientific community has devoted significant effort towards designing, synthesizing and testing a broad range of silicon–carbon composite structures with the main goal of improving charge transport. While the choice of carbon is a logical one as the material is abundant, inexpensive and easily processable, the available design parameter space is extremely large. Despite the many promising reports, more investigation is still necessary to fully stabilize silicon nanostructures and make them compatible with real-life battery operation requirements. In particular, the optimization of carbon coatings alone is likely not to be sufficient to achieve good functionality. Binders and electrolyte chemistries specifically optimized for silicon are also necessary, as it will be discussed in the next two sections.

### 2.3 Binders

Environmental and safety concerns have pushed the battery industry to replace *N*-methyl-2-pyrrolidone (*N*MP) as solvent/dispersant with water, bringing additional economic benefits because of the elimination of expensive solvent recovery equipment. This has also led to the replacement of poly(vinylidene fluoride) (PVdF) which is a mutagenic and teratogenic binder with carboxymethyl cellulose (CMC) and styrene butadiene rubber (SBR).^79^ These are now the state-of-the-art binders for commercial graphite electrode industrial production. Binders are crucially important when it comes to enabling long cycle stability in any battery material. The case of silicon is particularly interesting since swelling upon lithium insertion puts additional “stress” on the binder, which now needs to maintain the structural integrity of an anode undergoing very dynamic changes during charge–discharge. An early report from Lestriez *et al.*^80^ confirms that the standard workhorse binder for the case of graphite, which is poly(vinylidene fluoride) (PVdF) is not the optimal choice for silicon. Instead, superior performance is observed when using sodium carboxymethyl cellulose (CMC) as binder, which performs better than other tested chemistries (CMC with the addition of poly-ethylene-*co*-acrylic acid, polyvynilpyrrolidone, and PVdF). The authors test the binders by mixing 1–5 μm silicon powders with Super P carbon black and the binder in the 1–2% range by weight. It is interesting also to point out that the authors report a significant dependence of performance over pH of the water-based slurry, with lower pH improving capacity retention. They attribute that to cross-linking of the CMC chains, resulting in a more structurally stable assembly after coating. Hochgatterer *et al.*^81^ confirm the better performance of CMC for the case of mixed silicon–graphite anodes and provide additional insights into the reasons for this improvement. The authors first replace the carboxymethyl group with either hydroxyethyl or cyanoethyl groups, and clearly observe faster capacity fade. They then performed detailed FTIR analysis suggesting that a condensation reaction occurs at the interface between the oxide-terminated silicon particles and the CMC. In water, the sodium cation is partially substituted with H^+^ which interacts with the silicon surface hydroxide groups *via* hydrogen-bonding. Upon drying and dehydration, the polymer molecule is covalently bound to the silicon surface. The authors conclude that the good performance of CMC is due to this robust surface binding to silicon. Consistent with this explanation, CMC performs slightly better when the degree of substitution (DS) is higher (from 0.8 to 1.4). Magasinski *et al.*^15^ propose the use of polyacrylic acid (PAA) as a viable alternative to PVdF and CMC. The authors report that both CMC and PVdF can withstand a stress between 30 and 40 MPa at failure, although CMC elongates only 6% before failing while PVdf elongates 50%. PAA is stiffer than CMC, with negligible elongation and a failure stress of 90 MPa. Despite its brittle behavior, PAA leads to improved performances in silicon-dominant anodes, although the improvement is marginal compared to the already good performance of the CMC-based anode. Interestingly, the author perform their tests on particles with both a native oxide layer or coated by carbonizing a polycarbonate shell, and observe a slight improvement for the carbon-coated particles. The authors attribute the improved performance of PAA to its higher density of carboxylic groups, *i.e.* the groups that anchor securely to the particle surface. Promising performance is also reported by Kummer *et al.*,^82^ who directly polymerize aniline at the surface of ∼40 nm silicon particles creating a highly conformal polymer shell. These reports suggest that binder chemistry has a profound influence on silicon anode performance.

**Fig. 7:**
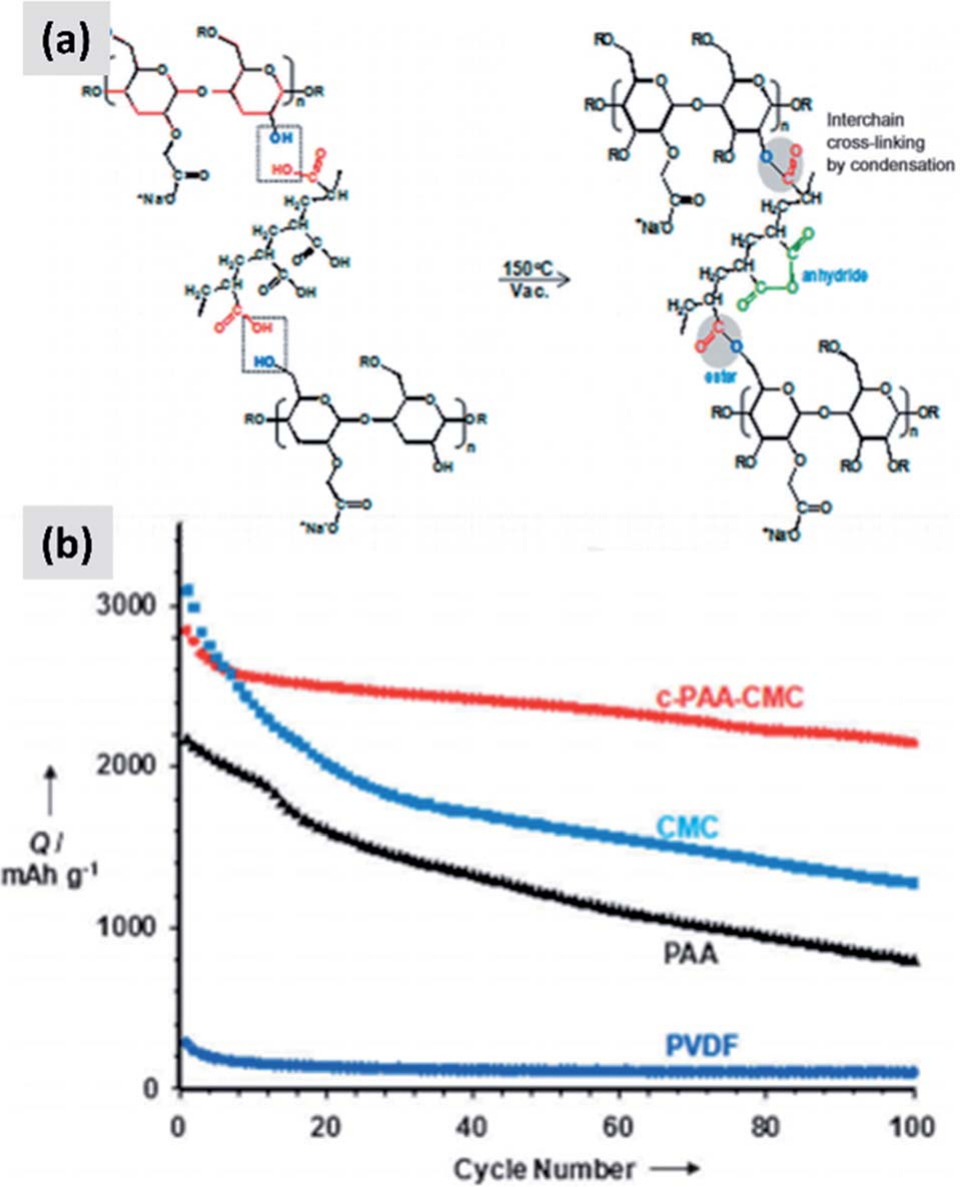
(a) Schematic showing the cross-linking chemistry between PAA and CMC. (b) Capacity of a silicon-based anode comparing the performance of PVDF, PAA, CMC and cross-linked PAA–CMC binders. Reproduced with permission from ref. 83.

Common polymers such as PAA and CMC may not be sufficient alone to stabilize silicon over many cycles. For that reason, several groups started investigating more complex binder chemistries and developing binders which are precisely tuned to the needs of silicon.^83–86^ For instance, Koo *et al.*^83^ report a process in which both CMC and PAA are utilized. After coating a slurry containing both polymers, the film is cured at 150 °C to cross-link the two polymers and realize a binder that is strongly attached to the silicon particles (*via* the abundant carboxylic acid in PAA) and at the same time can tolerate the volume changes of the anode (*via* the higher tolerance of CMC to elongation). The authors show conclusively that their approach provides improvements compared to the PAA-only and CMC-only case (Fig. 7). Silicon nanoparticles with a native oxide layer were used for their study. A similar concept is proposed by Song *et al.*^84^ who combine PAA with polyvinyl alcohol (PVA). For this study, particles with sizes between 30 and 100 nm and with a native oxide were used. After coating and drying of the water-based slurry, a thermal treatment at 150 °C for 1 hour is used to cross-link the two polymers. The authors report excellent stability, with high first cycle CE and ∼70% capacity loss only after 300 cycles, for an areal loading exceeding 4 mA h cm^−2^.

Binders also play a crucial role in determining the manufacturability of the formulation, as they affect parameters such as slurry viscosity and degree of sedimentation. These in turn affect the coating quality and the anode stability. Yim *et al.*^85^ compare binders such as PAA, CMC, PVdF and poly(amide imide) (PAI) and find that while PAA has the best mechanical properties in term of withstanding high stress, it also leads to substantial sedimentation in water-based slurries when using 50 nm silicon particles. The author solve this issue by cross-polymerization with PAI. Nguyen *et al.*^86^ explore the use of poly(acrylic-*co*-maleic) acid (PAMA) as a dispersant and find it to be efficient at preventing the sedimentation of 150 nm silicon particles and at dispersing the carbon black conductive additive, although they also find that excessive use of PAMA is detrimental. The attribute that to competitive binding to the silicon surface with CMC, *i.e.* the dispersant diminishes the effectiveness of the binder. This issue can also be alleviated by mixing the slurry components in the appropriate order (*i.e.* by mixing CMC and silicon particles before adding the dispersant). In addition, the authors^86^ also find that the addition of styrene-*co*-butadiene rubber copolymer (SBR) is highly beneficial to improve adhesion to the copper foil. These contributions confirm that there are many subtleties associated with the fabrication of battery anodes. Maximizing functionality and manufacturability likely involves developing complex formulations, with multiple binders acting in synergy and additives (dispersants and surfactants) to improve coating quality.

Several groups have invested time into developing more complex binder chemistries, often leveraging the knowledge accumulated in other fields with respect of conductive polymers (organic electronics).^87–91^ For instance, Wang *et al.*^87^ discuss the use of a self-healing polymer (SHP) as a binder for micron-size (3–8 μm) silicon particles. The SHP concept is based on the abundant hydrogen-bonding sites that can effectively repair the polymer and maintain its binding effectiveness as the silicon particles undergo continuous volume changes.^88,89^ The authors^87^ report that the polymer is highly stretchable and conductive over several cycles, and observe a remarkable improvement compared to CMC. They report >80% first cycle CE, with 4000 mA h per gram capacity for over 100 cycles and an areal capacity of ∼2 mA h cm^−2^. Park *et al.*^90^ design, synthesize and test electronically-conductive polymers based on the well-known concept of π–π stacking. Essentially, they graft pyrene groups onto a methacrylate backbone, realizing either poly(1-pyrenemethyl methacrylate) (PPy) or poly(1-pyrenemethyl methacrylate-*co*-triethylene oxide methyl ether methacrylate) (PPyE). They find that PPyE performs better because of its stronger interaction with the surface of the silicon particles. When tested with ∼100 nm nanoparticles, the anode shows ∼2000 mA h per gram capacity for 1000 cycles with ∼70% first cycle CE. Wang *et al.*^91^ have recently discussed the performance of a conductive glue based on the copolymerization of poly(3,4-ethylenedioxythiphene):polystyrene sulfonate (PEDOT:PSS) with vinyl acetate-acrylic (VAA). PEDOT:PSS is ubiquitous in the field of organic electronics and well-known for its good charge transport properties. The simple copolymerization procedure leads to outstanding results. In combination with ∼100 nm silicon particles (with native oxide termination), the conductive glue can enable high capacity (∼2000 mA h per gram) for almost 1000 cycles with >80% first cycle CE and most importantly at an areal loading of 5 mA h cm^−2^.

One important consideration that determines the binder selection is its interaction with the electrolyte and its effect on SEI growth. Nguyen *et al.*^92^ discuss this in details by performing FTIR and XPS analysis on electrodes that utilize PVdF, CMC and PAA as binders. For the case of PVdF, the authors observe a strong signature from lithium alkyl carbonates groups (ROCOOLi) and lithium carbonate (Li_2_CO_3_), similar to the case of graphite. For the case of PAA and CMC, the carboxylic acid group is replaced upon cycling with lithium carboxylates (–COOLi). Moreover, the growth in signal from the carbonate groups is significant, over the first few cycles, for the case of PVdF, while it is comparatively slower for PAA and CMC. The authors conclude that the reaction products between PAA, CMC and the electrolyte reduce the continuous decomposition of the electrolyte, leading to a thinner and more stable SEI. This is an important point, proving that the abundant carboxylic groups present in CMC and PAA not only help anchoring to the silicon surface, but also passivate the surface in such a way that a thinner SEI layer is achieved, reducing capacity fade.

### 2.4 Interfacial chemistry

The interaction with the electrolyte is crucially important for any battery material. During the first lithiation cycle, decomposition of the electrolyte occurs resulting in the formation of a solid electrolyte interphase (SEI) which for the case of ethylcarbonate/diethylcarbonate (EC/DEC)-based electrolyte is composed of a mixed organic–inorganic layer that irreversibly traps lithium ions upon formation. Ideally the SEI growth is self-terminating. A stable SEI allows for lithium diffusion into the active material and prevent lithium plating at the surface of the active material, meaning that it is necessary for the stable operation of the battery. Early reports confirmed that the SEI chemistry and formation mechanism is different in the case of silicon compared to graphite.^93,94^ Oumellal *et al.*^93^ perform a combination of cyclic voltammetry, impedance spectroscopy and nuclear magnetic resonance (NMR) on silicon anode with a CMC binder. They observe a continuous decrease of the low-voltage discharge peak which eventually drops from 0.2 V to the cutoff voltage 0.05 V. This is accompanied by an increase in the overall impedance of the anode. They interpret these observations as a signature of a continuously growing SEI layer which progressively prevents lithium ion from accessing the active materials, resulting in capacity fade. Similar results are reported by Michan *et al.*^94^ with the addition of cross-sectional SEM analysis. They observe an effective densification of the nanoparticle-based coating, resulting from the continuously growing SEI layer. They point out that SEI exfoliation from the particles is likely to occur as well. This increases the effective tortuosity of the coating and affects the lithiation kinetics, with lithium ions unable to access the whole nanoparticle layer. The use of additives to electrolyte solutions such as vinylene carbonate (VC) was already well-known to improve the battery performance even for the case of graphite anodes. Aurbach *et al.*^95^ for instance reports that the addition of VC (5% by volume) to a ethylene carbonate–dimethyl carbonate (EC–DMC)–lithium hexafluoroarsenate (LiAsF_6_) electrolyte leads to a thinner and more cross-linked SEI layer compared to the a regular formulation (without VC). Chen *et al.*^96^ test the effect of VC on silicon for the case of thin film sputtered on copper foil. The anode show a considerable improvement in stability and increase in coulombic efficiency upon addition of VC. SEM analysis shows that the SEI layer is smoother and thinner when VC is added, and impedance spectroscopy confirms that the overall impedance is stable over the first few cycles, whereas it increases considerably for the VC-free case. Choi *et al.*^97^ use a very similarly approach, *i.e.* focusing on silicon thin films, but using fluoroethylene carbonate (FEC) in place of VC. Their observations are similar as to the case of VC, meaning the addition of FEC (3% by weight) leads to a smoother and thinner SEI. XPS analysis also shows a stronger signature from lithium fluoride (LiF) when FEC is added. Again, FEC significantly improves anode cycling stability. Nguyen *et al.*^98^ compare the effect of FEC and VC for the case of nanoparticle-based anodes. The formulation is based on 500 nm silicon particles using a mixture of CMC and PVA as binder. The authors carefully vary the amount of VC and FEC in their electrolyte, and present a wealth of data including cyclic voltammetry, impedance spectroscopy, SEM, FTIR and XPS. Electrodes cycled without FEC or VC have a SEI which is mainly composed by lithium alkylcarbonates and lithium carbonates. The use of VC leads to the appearance of poly(VC) and LiF at the surface. FEC behaves similarly to VC, although the presence of poly(FEC) species is observed at a high level of addition (25% by volume in the electrolyte). Impedance spectroscopy shows that at such a high level of FEC addition, the overall impedance increases to the point that FEC is detrimental to performance. The authors therefore recommend the use of FEC at a 10–15% concentration by volume, since this helps stabilizing the SEI with a beneficial effect to stability. FEC is found to be more effective than VC from an overall anode impedance point of view. Additional insights are provided by Sina *et al.*^99^ who present extensive electron energy loss spectroscopy (EELS) measurements on silicon particles cycled with and without FEC addition to the electrolyte. Consistent with previous result, the authors find that LiF is predominantly formed when FEC is added. They also find that FEC effectively prevents the formation of lithium silicates, which are expected to be detrimental to the anode impedance. The authors present data after the first and after 100 charge–discharge cycles. After many cycles, the surface chemistry is very similar for the case in which FEC is added compared to the FEC-free electrolyte, consistent with the fact that FEC is likely consumed in the first few cycles and converted into a mechanically stable LiF layer in close contact to the particle surface.

**Fig. 8:**
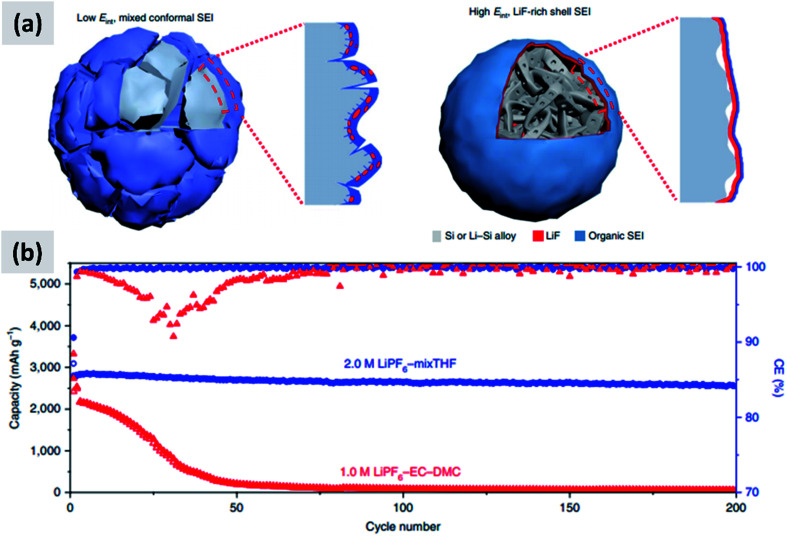
(a) Representation of the SEI formed with the regular electrolyte (left) and the one obtained using a THF-based solvent. (b) Comparison in cycling performance for the two electrolyte chemistries. Reproduced with permission from ref. 17.

These reports confirm that the understanding of SEI formation and its influence on silicon anodes performance has advanced significantly. Recent efforts have focused on designing and engineering an artificial SEI layer, with the goal of helping the stabilization of silicon over several cycles. Li *et al.*^100^ apply a lithium phosphorous oxynitride (LiPON) *via* sputtering on top a silicon thin film. LiPON is a well-known lithium-ion conductor, and it presence indeed helps stabilizing the performance of the structure over 100 cycles. Most importantly, the artificial SEI dramatically improves the coulombic efficiency of the battery. Jin *et al.*^101^ discuss the design of a TiO_2_–silicon yolk–shell structure in which the TiO_2_ shell is not perfectly continuous. As a result, the SEI layer eventually forms in the buffer space between the silicon core and the titania shell. This geometric constraints stabilizes the SEI. Finally, a particularly interesting report is the recent one from Chen *et al.*,^17^ in which the EC–DMC co-solvent is replaced by a mixture of tetrahydrofuran and 2-methyltetrahydrofuran. The lithium salt is the commonly used LiPF_6_, at 2 M concentration. The authors find that LiPF_6_ dissolved in the mixed THF solvent has a higher reduction potential compared to the EC–DMC case. As a consequence, the decomposition of the salt occurs before that of the solvent in the formation cycle, as opposed to the EC–DMC case in which the react simultaneously at the anode surface. The result, as confirmed by detailed XPS analysis, is an SEI which consists of an inorganic–organic bilayer with LiF in contact with the silicon surface and the polymerized carbon in the outer layer. The authors argue that LiF is mechanically robust and has a high interfacial energy with silicon and silicon–lithium alloys, preventing its fracture during the continuous volume changes of the particle. As a demonstration, the authors test this concept with micron-size silicon particles (10 μm) and observe outstanding performance with ∼2800 mA h per gram capacity (5.6 mA h cm^−2^), a 90% drop in capacity at 400 cycles at C/5, a 90% first cycle CE and a coulombic efficiency reaching 99.9% after only 7 cycles. These results are summarized in Fig. 8. The SEI chemistry is crucial and it has been comparatively less studied than nanostructuring of silicon, making this area particularly interesting for future investigations.

## 3. Silicon nanoparticles: processing-properties relations

Despite few promising reports on the use of micron-sized silicon particles as anode material, the vast majority of researchers focus on nanoparticles, as it is well accepted that reduction in size is a viable approach to overcoming the pulverization issues associated to lithiation.^28^ As extensively discussed earlier in this manuscript, nanostructuring solves one issue (mechanical stability) but introduces several other ones (handling of volume changes, charge transport through coatings composed of fine particles, large surface-to-volume ratio). An important aspect that has been somewhat overlooked by the community is the optimization of the nanoparticle properties in terms of size distribution. This is strongly affected by the synthesis approach, and has important consequences on material cost and its potential for commercialization. In this section we first provide evidence supporting that particle size and size distribution has a strong influence of performance. We then discuss various silicon nanoparticle synthesis techniques, highlighting strengths and weaknesses of each approach and potential for large-scale utilization.

### 3.1 Size distribution and its influence on anode materials

**Fig. 9:**
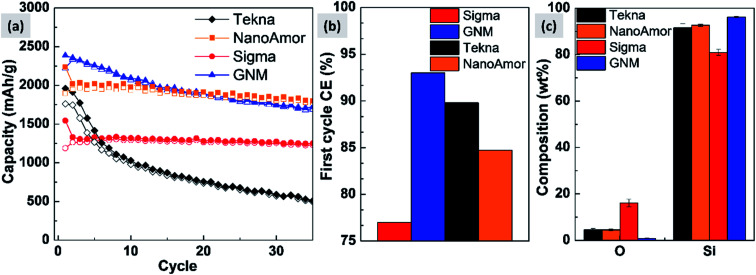
(a) Comparison of cycling stability between commercially available silicon nanoparticles. This was performed in half-cells, with different starting powders processed and tested in exactly the same conditions. (b) First cycle coulombic efficiency for the four powders under consideration. (c) Elemental analysis for the tested commercially available powders.

**Fig. 10:**
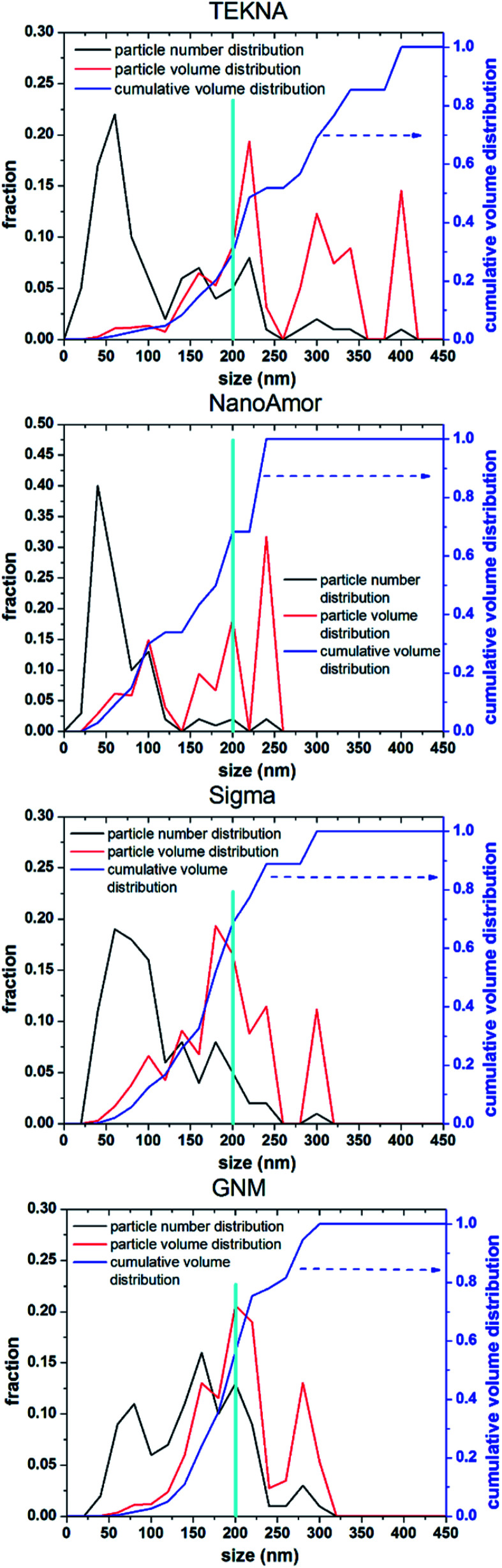
Number (particle) size distribution, volume distribution, and cumulative volume distribution (on the left axis) for commercially available silicon nanoparticles. We highlight a size of 200 nm to guide the eye in comparing the distributions.

In Fig. 9 we show the performances of various commercially available particle, as tested in a silicon-dominant anode. We select particles from Tekna (distributed by Sigma Aldrich, product number 795585), Sigma Aldrich (product number 633097), Nanostructured & Amorphous Materials (abbreviated NanoAmor, product Si 100 nm) and GetNanoMaterials (abbreviated GNM, product Si-111), since these are readily available and the most commonly utilized in the scientific literature. These particles are all marketed as having sizes around 100 nm. The particles are coated with a graphitized carbon shell following the procedure described in ref. 75 and tested in half cells with CMC as binder and 10% FEC addition to a standard EC/DEC electrolyte formulation. The direct comparison between these materials, all nominally having the same size, shows that there are major differences in performance with respect of stability. Fig. 9a shows that the powders from Tekna and GNM show a faster fade in capacity compared to those from Sigma and NanoAmor. The first cycle coulombic efficiencies for these materials are shown in Fig. 1b. Again there are important differences among these powders. The material from Sigma has the lowest CE (∼76%), which the powder from GNM shows an impressive value of 93%. Elemental analysis is shown in Fig. 9c. All powder have a relatively low degree of oxidation, with the exception of the sample from Sigma. This correlates with the low first cycle CE for this sample, and confirms that the amount of oxidation should be minimized. Nevertheless, the powders from GNM, Tekna and NanoAmor show very different behavior in a half-cell, despite being nominally similar in size. To further investigate this behavior, we have performed careful characterization of the size distribution by analyzing several TEM micrographs of these commercially available samples. The particle size distributions for each sample are shown in Fig. 10. We also show the volume distribution, *i.e.* the fraction of the total volume occupied by particles with a given size. The cumulative volume distribution is shown as well. The average particles sizes are 110 nm for TEKNA, 95 nm for Sigma Aldrich, 60 nm for Nanostructured & Amorphous Materials, and 140 nm for GNM. The broader size distribution of the samples from Tekna and GNM correlate with their faster capacity decay. The cumulative volume distribution is particularly useful. Only 20% (TEKNA) and 50% (GNM) of the volume is occupied by particles smaller than 200 nm. Assuming that the material density is constant as a function of size, which is a safe assumption for these size ranges, the cumulative volume distribution provides an indication of the fractional mass of material below a given size. When a significant volume is occupied by large particles, pulverization and capacity fade becomes dominant. The samples from Sigma and Nanostructured & Amorphous Materials have both around 70% of the volume at sizes below 200 nm, consistent with the higher stability. Most importantly, it suggests that simply considering the average particle size when selecting a source of silicon nanoparticles is not appropriate, since this application is particularly sensitive to the tail of the size distribution towards larger particles. Even a small fraction of large particles implies that a large volume and mass fraction is occupied by structurally unstable material, which is conducive to poor performance. This analysis also confirms that proper selection of silicon nanoparticles is critical for developing an anode that can meet the requirements of commercial operation. Minor differences in size distribution and oxidation level can make a significant difference in capacity fade and coulombic efficiency. The production process determines the size and size distribution of the nanoparticles, and different production techniques will be discussed in the next sub-section.

### 3.2 Milling

Ball-milling involves loading chunks of materials into an inert vessel typically composed of hard materials (hardened steel, carbides). The vessel is agitated vigorously, often in presence of a milling media that help with breaking down the material into smaller and smaller powder. The power input in such processes is quite considerable, on the order of 200 W for a typical sample size of 10 grams.^102^ Bulk materials can be pulverized to a very small size, of the order of few hundreds of nanometer, although extensive milling times of the order of several hours are needed to achieve such size range. Still, there are appealing aspects to this process. First, it does not use any chemical precursors, but rather uses bulk materials as starting point. This is particularly interesting for silicon, since it would allow using low-cost grade such as metallurgical silicon, or use the waste from the solar cell industry (known as silicon kerf). In addition, ball milling is ubiquitous in the powder processing industry, therefore it is a known and trusted process. Finally, the amount of energy input is so significant that it is possible to structurally and chemically modify the material during milling.

Shen *et al.*^103^ use milled silicon particle to realize a yolk–shell structure analogous to the one described by Liu *et al.*^11^ The authors use metallurgical grade, polycrystalline and kerf silicon as precursor. Milling for 5 hours leads to 100–200 nm silicon particles with irregular sizes. Anodes developed using the raw powder have poor performance, while the yolk–shell approach greatly stabilizes the material, with a drop in capacity by a factor of 2 only after 1000 cycles at 1C. No significant differences in performance are observed between the 3 different types of silicon source. Even simpler strategies have been proposed by other research groups.^104–106^ For instance, Liu *et al.*^104^ prepared silicon–carbon composited by pyrolysis of poly(vinly chloride) (PVC) on micron-sized silicon particles, followed by high-energy ball milling and another PVC coating and pyrolysis step. The resulting materials is a silicon–carbon composite with particle size in the range of few tens of microns. XRD of the resulting material shows a clear signature from graphitic carbon. The reported anode performance is promising, with reasonably high (∼80%) first cycle CE and good charge–discharge stability. The authors obtain outstanding stability by limiting the discharge capacity at 600 mA h per gram. Unfortunately the corresponding volumetric energy density is not reported. A similar approach is proposed by Datta and Kumta^105,106^ using either polyacrylonitrile (PAN) or poly[(*o*-cresyl glycidyl ether)-*co*-formaldehyde] as carbon precursor. Moreover, the authors using high-energy ball mill to mix silicon with graphite before pyrolysing the organic matrix and converting it into an amorphous carbon that securely binds the silicon to graphite. The silicon weight fraction for these composite is around 30%. The authors report good stability over few tens of cycles, first cycle CE exceeding 70%, and a capacity of 600 mA h per gram. Unfortunately the coulombic efficiency after the first few cycles is not mentioned in these studies, and it would have provided some useful indication of the potential of this approach.

To summarize, ball milling is attractive with respect of ease-of-operation and potential for providing low-cost silicon powders, and it can also be utilized to realize uniformly mixed composites. Its limitation is in the fact that it cannot readily provide nanoscale free-standing particles with good control over size distribution. Most of the community has focused on techniques that can supply nanoscale-silicon, given the important of nanostructuring in the stabilization of silicon during lithiation. Therefore milling has not attracted as much interest as other production techniques, although the work summarized in this section indicates that it may be possible to develop commercial-grade silicon-containing anode materials based on milled powders.

### 3.3 Gas-phase techniques

The gas-phase synthesis is the predominant approach for the production of silicon nanoparticles. This class of processes is very diverse. Most of the technique use silane (SiH_4_) as precursor, but there are several options with respect of how to activate the molecule and drive the formation of nanoparticles. This can be done by heat, laser or by generation of a plasma (*i.e.* an ionized gas). While there are important difference among these techniques, as it will be described in this sub-section, they share the capability of producing small particles (tens to hundreds of nanometers), with high yield and with excellent precursor utilization rate. This last point is critical, since silane is considerably more expensive that the bulk silicon used for milling. However, options are available for the gas-phase conversion of other precursors (like chlorosilanes or even silicon powders) to silicon nanoparticles.

**Fig. 11:**
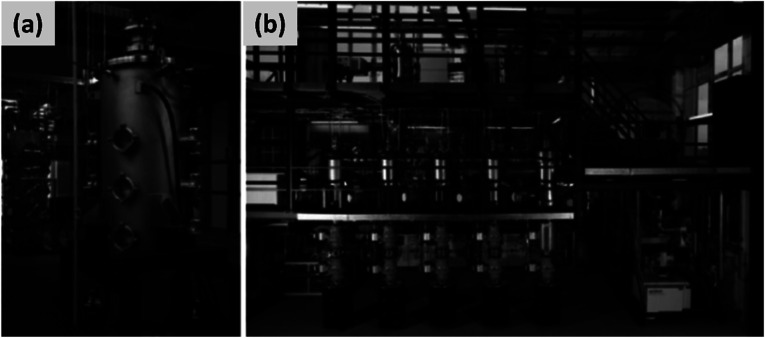
(a) Photograph of a pilot-scale hot wall reactor and of its related plant (b). Reproduced with permission from ref. 110.

The onset of thermal decomposition for silane occurs around 400 °C. It is therefore relatively straightforward to use a heated tube furnace to de-hydrogenate the precursor and drive the formation of silicon powders. Wu *et al.*^107^ provide an early example of this process by flowing silane in a multi-zone tube furnace in which the temperature is progressively increased from 500 °C to 1200 °C. The authors report the production of crystalline silicon particles with an average size of 150 nm, as measured in-line *via* an aerosol sizer. While the authors do not report the particle mass yield or the efficiency with which the precursor is utilized, it is well-known that diffusional losses to the reactor walls are the main loss mechanism in such systems. The diffusion coefficient for small particles (*i.e.* with diameter smaller than the gas mean free path) increases linearly with temperature and varies as *D*_p_^−2^, with *D*_p_ being the particle diameter. Consequently, small particles are easily lost in hot-wall reactor, especially at the early stages of growth. Alam and Flagan^108^ describe a modified version of the process in which a seed aerosol of silicon particles is generated in a first hot-wall reactor, then injected into a second hot-wall reactor to which additional silane precursor is added. By carefully controlling the temperature in the second reactor (around 500 °C) it is possible to grow the seed particles to larger sizes *via* chemical vapor deposition while avoiding the nucleation of new particles. The authors obtain particles with an average size around 10 μm, with full conversion of the silane added to the second stage into silicon particles. Wiggers *et al.*^109^ describe a scaled-up hot wall reactor with an inner diameter of 70 mm and operating at 1 atm, at a temperature of 1000 °C. The author use a standard flow rate of ∼10 slm (standard liters per minute) of up to 40% silane in argon. The residence time in the hot region, which is 1 meter long, is between 2 and 3 seconds depending on process parameters. Up to 92% of the silane input is converted into particles. The produced particles appear as fractal agglomerate of ∼100 nm crystals. The same reactor is utilized by Kessler *et al.*^110^ to develop silicon-based thermoelectric devices. The authors report a production rate in the 0.5–1 kg per hour. A photograph of the pilot-scale reactor is reproduced in Fig. 11. The fractal agglomerates have a surface area of approximately ∼16 m^2^ per gram. These particles have been used as feedstock for several studies on silicon anode development. For instance, Kummer *et al.*^82^ investigated the use of polyaniline as a conductive additive, and Xiao *et al.*^111^ combined the silicon particles produced *via* thermal pyrolysis of silane with carbon nanotube and graphene.

**Fig. 12:**
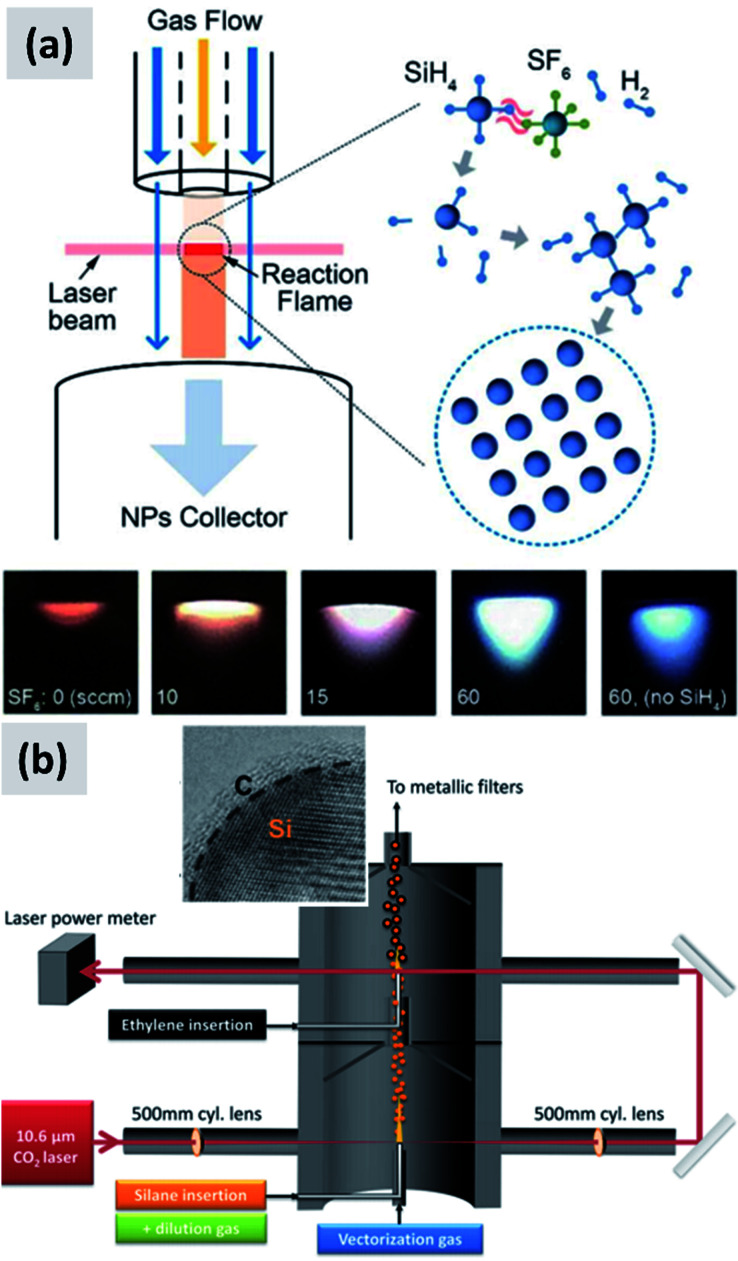
(a) Schematic of a silane laser pyrolysis process in which SF_6_ is added as photosensitizer, with the goal of fully reacting the silane gas. Photographs of the laser-generated flame are also shown for different levels of SF_6_ flow. Reproduced with permission from ref. 115. (b) Schematic of a two-steps laser pyrolysis scheme for the synthesis of silicon nanoparticles and their in-flight coating with a carbon shell. Reproduced with permission from ref. 116.

In laser-driven pyrolysis the decomposition of silane is driven by the localized delivery of energy at the focal point of a laser beam. Typically, a CO_2_ laser is used for this application because of the efficient absorption of silane at 10.591 μm. One crucial difference compared to hot-wall reactors is the fact that the energy delivery is strongly spatially localized, generating steep gradients in temperature that can drive the rapid nucleation of particles therefore minimizing diffusional losses. Cannon *et al.*^112,113^ provide some of the early demonstration of this process as applied to the case of silicon. They use a 150 W CO_2_ laser and estimate that roughly 23 W of the input power is absorbed by silane. They report an electrical-to-optical power conversion efficiency of roughly 15% for such lasers. The reactor is run at a pressure between 0.2 and 1 atm. The authors use a silane flow rate of 11 sccm (standard cubic centimeters) per minute, and find that the precursor is fully depleted when passing through the laser beam. This results in ∼50 nm silicon particles with a specific surface area of ∼60 m^2^ per gram and a yield of 1 gram per hour. Further tuning of the process parameters allows controlling particle size over a broad range, enabling the high-yield production of sub-10 nm particles,^114^ and making the tunability of this process one of its desirable properties. Kim *et al.*^115^ describe adding sulphur hexafluoride (SF_6_) to the gas stream as a photosensitizer, *i.e.* with the goal of increasing the absorption of laser radiation by the gas stream (see Fig. 12a). This translates into an improved utilization of silane, virtually approaching 100%. The produced particles have an average size around 20 nm. No contamination from sulphur or fluorine is detected, confirming that SF_6_ is optically active but chemically inert in this process. Sourice *et al.*^116^ describe a novel strategy for the laser-driven synthesis of silicon nanoparticles and their in-flight coating with a carbon shell. A CO_2_ laser beam is first passed through a silane stream to nucleate ∼20 nm silicon crystals. Mirrors are then used to redirect the beam into the aerosol a second time, after ethylene is added to the gas stream, as shown in Fig. 12b. This results into the in-flight coating of the silicon particles with a 2–3 nm thick amorphous carbon shell. The authors report a large increase in specific surface area after the in-flight coating, increasing from 66 m^2^ per gram to 156 m^2^ per gram, suggesting that the carbon shell is porous. The authors also report that a fraction of the ethylene is converted not in a conformal carbon coating but in free-standing carbon particles. So while interesting, this study suggests that careful control of the process parameters (precursor concentration, laser fluence) is needed to realize the desired structure. Nevertheless, the authors observe a clear improvement in anode performance with respect of both charge stability and coulombic efficiency. The prospect of the direct and continuous growth of a carbon protecting layer around the silicon particles is particularly appealing, since it allows streamlining the manufacturing process and increasing yield considerably (*i.e.* decrease the overall processing time).

**Fig. 13:**
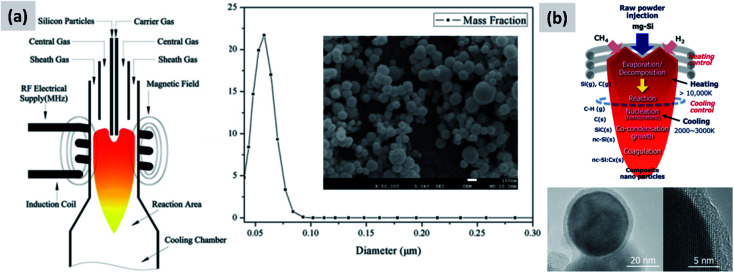
(a) Schematic of a RF inductive thermal plasma reactor for the conversion of micro-sized silicon particles into silicon nanoparticles. The representative mass distribution as a function of size is shown, together with a SEM image of the produced material (scale bar is 100 nm). Reproduced with permission from ref. 127. (b) Schematic of a thermal plasma process for the conversion of micron-sized silicon particles into silicon particles and their in-flight coating with a carbon shell. Representative TEM of the core–shell structure is also shown. Reproduced with permission from ref. 118.

Plasmas have also been extensively used to supply silicon particles to groups actively researching silicon-based anodes.^58,59,117–120^ The term plasma refers to an ionized gas and covers a very broad range of systems. In general, plasmas can be categorized as thermal (*i.e.* all components of the system are close to having the same temperature) and non-thermal (*i.e.* free electrons are significantly hotter than the background gas). Thermal plasmas provide an intense and localized heat source, capable of producing nanoscale silicon particles with at a very high processing rate. The fundamentals of particle growth in these systems has been carefully described by Girshick and Chiu:^121^ the gas temperature in the plasma is typically around 1 eV (∼10 000 K) and sufficient to fully dissociate any molecular precursor. As the gas leaves the plasma volume and cools down, the precursor vapor reaches a sufficiently high super-saturation ratio to drive the rapid nucleation of small (sub-micron) particles *via* homogeneous nucleation. Rao *et al.*^122^ describe a thermal-plasma-based process for the synthesis of silicon particles. It relies on a DC plasma torch operating in an argon–hydrogen mixture lose to atmospheric pressure, with a power input of 4 kW. Silicon tetrachloride (SiCl_4_) is vaporized and injected in the exit plume of the plasma torch. The gas is then expanded though a nozzle, during which rapid gas cooling and the nucleation of particles occur. The authors report the formation of ∼10 nm silicon particles with a lognormal size distribution and a geometric standard deviation of 1.6. This implies that all particles are effectively below 50 nm in size. While the production rate for this system is not reported, it is interesting to highlight the use of a chlorinated silicon precursor (SiCl_4_) as opposed to the use of silane (SiH_4_). SiCl_4_ is considerably less expensive and easier to handle compared to silane, as it is a liquid as opposed to a pyrophoric gas. This offers a pathway towards reducing the cost of gas-phase produced silicon particles. On the other hand, Dogan *et al.*^123,124^ have investigated the use of a remote thermal plasma system to grow silicon nanoparticles from silane. The precursor is injected downstream of the plasma volume, where the gas and electron temperature is relatively low (0.1 eV). For that reason, the authors propose an ion-driven particle nucleation process which results in the full utilization of silane and in the formation of ∼80 nm silicon nanocrystals. The power consumption is 1–2 kW h, the silane flow rate is as high as 600 sccm, and the production rate is very high and equal to 6 grams per hour. The use of an inductive coupling, as opposed to the DC systems used in the papers above, enables a significant increase in plasma power and density.^125^ The inductively coupled, radio-frequency thermal plasma provides a very intense heat source that can process metal, semiconductor or ceramic materials. Guo *et al.*^126^ report the synthesis of sub-micron silicon particles using a ∼15 kW commercial inductive torch (from Tekna) and micron-sized silicon powder as precursor. A powder feeder is used to deliver silicon powders to the intense plasma volume at a rate as high as 5 grams per min. The powder is fully vaporized by the plasma. Nucleation of submicron silicon crystals occurs as the vapor exits the plasma volume. He *et al.*^127^ provide a detailed description of the process. They use a 30 kW RF induction torch (schematic shown in Fig. 13a) to produce sub-micron silicon particles starting from 15 μm or 30 μm silicon powders. They find that the residence time in the plasma is around 20 ms, which is sufficient to fully vaporize the 15 μm particle but not the 30 μm particles. As a result, the particles produced from the 15 μm powders have an average size around 60 nm (also shown in Fig. 13a), while those produced from the 30 μm powder have a bimodal distribution, with a significant fraction of particles as big as 300 nm. Liu *et al.*^117^ use a very similar process to produce silicon particles of varying sizes and test them as an additive to graphite-dominant anodes. The author use micron-sized silicon powders as precursor. These are fed to a 15 kW induction plasma torch at a rate of 1 gram per min. The authors use nitrogen as an inert quenching gas to controllably cool the gas stream exiting the plasma volume, and thus tune the particle nucleation rate and size. The authors report more stable performance when 15 nm silicon particles are used as additive as opposed to 70 nm. No special technique is used to passivate or protect the silicon particle surface. Still, this report confirms that it is possible to achieve a precise control over particle size when using the RF induction plasma approach. Thermal plasmas systems can also be configured not only to grow silicon particles but also to modify their surface in-flight. Kambara *et al.*^118^ describe a 90 kW inductive RF torch system in which metallurgical grade silicon powders are used as precursor, with a feed rate as high as 6 grams per minute. Methane is added to the gas stream in the outer region of the reaction volume, as shown in Fig. 13b. The authors obtain <100 nm silicon particle with a conformal carbon coating, although they also report the formation of silicon carbide particles if the methane flowrate is too high. The material is tested in a silicon-dominant anode with promising results (65% first cycle CE, ∼1000 mA h per gram and 75% capacity loss over 100 cycles). While further improvements are needed to achieve commercially-compatible performance, the process is very promising as it starts from an inexpensive precursor (metallurgical grade silicon) and produces core–shell silicon–carbon particles in a single step, at a high production yield.

Non-thermal plasmas have also been extensively investigated as nanoparticle sources, with abundant literature on this topic.^128^ Gas temperature in these system is only slightly above room temperature. The presence of highly energetic electrons, with typical densities in the 10^10^ cm^−3^ range and temperatures in the 1–5 eV range, leads to the activation of gas-phase precursor and the formation of particles. For the case of silane, electron-induced abstraction of hydrogen initiates a polymerization reaction which eventually leads to the formation of nanocrystals.^129^ Lopez and Mangolini^130^ perform an extensive characterization of flow-through low-temperature plasma reactors for the conversion of silane into silicon particles. Characterization of the gas-phase *via* FTIR confirms that silane is consumed very rapidly in these systems (within few milliseconds) and that particles rapidly grow to a few nanometer in size (5–10 nm range). Interestingly, the non-thermal plasma approach effectively prevents further size growth because of electrostatic stabilization of the aerosol. This is induced by the lack of thermal equilibrium between electrons and heavy species (ions), with electrons rapidly charging the particles and electrostatic interaction preventing agglomeration. Yields as high as 1 gram per hour are achievable when using reactors with a 50 mm diameter, with an energy input of roughly 200 W at a 13.56 MHz excitation frequency. The convenient aspects of this process are its small footprint, small power consumption, and excellent silane utilization rate. Moreover, continuous flow non-thermal plasma reactors can be placed in series to modify the particle surface, *i.e.* with silicon particles grown in a first low-temperature plasma from silane and then aerodynamically injected into a second low-temperature plasma where the surface modification takes place. Yasar-Inceoglu *et al.*^120^ have demonstrated this for the case of polyaniline, which can be grafted conformally and in-flight onto silicon particles *via* the plasma-induced polymerization of aniline. Coleman *et al.*^131^ applied the same concept using methane as precursor in place of aniline, resulting in an amorphous carbon shell although care has to be taken to avoid carbonization and formation of silicon carbide crystals in the second plasma. These low-temperature reactors produce small particles whose surface can also be easily modified using well-established chemistry techniques to give stable colloidal dispersion.^132^ These have been used to realize large aggregates of small silicon particles, held together by carbon matrices obtained by various polymeric sources.^58,59^ The resulting structures show exceptional stability although they are marred by low first cycle CE. As discussed earlier, this is likely the consequence of irreversible lithium insertion into the glassy/amorphous carbon matrix. It is also important to stress that the synthesis of silicon particles in low temperature plasma reactors has been demonstrated when using chlorinated precursors such as SiCl_4_,^133–135^ opening a pathway towards reducing the cost of silicon particles produced *via* this route.

Gas-phase synthesis techniques are often considered as prohibitively expensive compared to others approaches such as milling. On the other hand, they can provide easy and reproducible access to small particles (tens to hundreds of nanometers), they are compatible with low-cost precursors such as metallurgical grade silicon powders and they can be operated continuously with a production rate of several grams per minute.

### 3.4 Magnesiothermic reduction

Silicon is industrially produced by reaction of silica with carbon, *i.e. via* a carbothermal reduction process. This process has become the industrially prevalent one because of the abundant availability of carbon coke as reducing agent. On the other hand, it has been known for decades that magnesium is also a viable reducing agent. For instance, Banerjee *et al.*^136^ already discuss in 1982 the use a magnesium-based method to reduce the silica contained in rice husks into high purity silicon, with the goal of reducing the cost of polycrystalline silicon and advance the utilization of photovoltaic panels. A comprehensive discussion of the process is presented by Zakaryan *et al.*^137^ The stoichiometric reaction SiO_2_ + 2Mg → Si + 2MgO is strongly exothermic, with an adiabatic combustion temperature of ∼1900 °C which greatly exceeds the melting point of silicon. The authors find that the reaction activation energy is considerably lower for the case of a solid–liquid reaction (*i.e.* silica reacting with liquid magnesium) than for a solid–solid reaction. This is consistent with the reaction onset being around 650 °C, *i.e.* at the melting point of magnesium. The reaction falls under the category of self-propagating high-temperature synthesis: once the reaction is initiated at 650 °C, the significant heat released by the oxidation of magnesium further increases the temperature and the kinetics of reaction, similarly to what occurs for the well-known thermite reaction between aluminum and iron oxide. The temperature can be controlled by varying the stoichiometry or by adding an inert compound such as sodium- or magnesium-chloride. This is also necessary to prevent the growth of the silicon particles into large crystal. The potential advantages of this technology include the fact that it uses relatively inexpensive precursors and its high rate. On the other hand, silicon can be extracted by the reaction products only *via* multiple etching steps in various acids, such as hydrogen chloride to remove Mg_2_Si, and hydrogen fluoride to remove oxides. This process can be tedious and generate large amount of hazardous waste.

The use of magnesiothermic reduction of silica has attracted great interest by many groups working on the development of silicon-based anodes.^138–143^ This process has the attractive property of being compatibles with the use of low-cost forms of silica, offering an opportunity to reduce materials' costs. Yoo *et al.*^138^ report the use of common sand for the development of anode materials. The authors use 300 μm sand, grind it to ∼1 μm in size *via* milling, then reduce it to silicon *via* a gas-phase magnesiothermic reaction. The resulting silicon powder is a hard agglomerate of small (few tens of nanometer) crystals. Carbonization of polyaniline at the surface of the powder results in material with ∼2500 mA h per gram capacity at a mass loading of ∼1 mg cm^−2^, with ∼80% capacity drop over 100 cycles. The first cycle CE is not reported. Wang *et al.*^139^ use diatomite, a biological sedimentary mineral, as starting point instead. This results in a highly porous silicon structure after magnesiothermic reduction. When coated with a carbon layer realized by carbonizing a phenolic resin, the material gives a good capacity (∼1600 mA h per gram) but a high rate of capacity fade. Xie *et al.*^140^ and Wang *et al.*^141^ use instead monodispersed, synthetic silica spheres around 300 nm in size as silicon source. Interestingly, upon reduction with magnesium and leaching of reaction byproducts the authors obtain ∼300 nm porous silicon particles. This suggests that the approach has the potential of controlling the porosity of the final structure, providing some buffer space to accommodate for the silicon volume changes. Both groups report very similar performance, with promising stability although the capacity loss over a few tens of cycles is still not compatible with a commercial application. The magnesiothermic approach is also viable when the silica precursor is already interfaced with a carbon-based component. For instance, Zhang *et al.*^142^ use a well-established polymer self-assembly technique to realize a mesoporous carbon–silica structure. Upon exposure to magnesium vapor, the authors obtain well dispersed silicon nanoparticles with few nanometers in size, dispersed into a periodic porous carbon matrix with pore size around 5 nm. The structure shows remarkable performance, with 88% first cycle CE and ∼1800 mA h per gram capacity, with excellent stability over 100 cycles. The areal capacity is 3.7 mA h cm^−2^. Unfortunately, the authors do not discuss the structure of the carbon matrix (graphitic *vs.* amorphous) or the actual volumetric capacity of the structure. Nevertheless, the stability provided by this material is among the best reported in the literature. Another interesting approach has been proposed by Wu *et al.*:^143^ the authors mix silica particles (∼300 nm in size) and graphene oxide to form an interconnected network. The magnesiothermic reduction step not only converts silica to silicon, but it also reduces the graphene oxide shell to graphene. The resulting structure shows good stability, although the first cycle CE is quite low (∼50%).

Overall, the magnesiothermic approach offers the advantage of utilizing silica, in its various forms, as low-cost precursor. The reports cited above confirm the compatibility of silicon produced *via* the magnesiothermic route with energy storage applications. A potential drawbacks is the effective production rate, since even relatively large batches (kilograms) still require extensive preparation time (such as milling of the silica and mixing with magnesium flakes) and post-processing time (leaching with acids). The generation of large quantities of hazardous waste is also an issue.

## 4. Summary and recommendations

The scientific community has made great strides towards engineering a silicon-containing anodes that can provide significant improvements in energy density. Silicon presents many challenges, and the numbers of proposals on how to address these issues is very large. The contributions discussed in this document are the ones that push forward the most promising approaches towards addressing these issues. There are four major conclusions that can be drawn from the overview of this field:

(1) Managing the volume expansion of silicon is crucial. This implies that the use of nanoparticles is necessary,^28^ although not sufficient. The most promising reports in the literature use some sort of nanostructuring in combination with designs that can accommodate for the volume change during lithiation, such as yolk–shell or porous structures.^11,12,29^

(2) Achieving high coulombic efficiency is necessary, especially when a silicon-based anode is interfaced with a real-life cathode material, *i.e.* when a large reservoir of lithium is not present. CE can be maximized by avoiding oxidation of the active material,^60^ utilizing high-quality carbon coating (as opposed to amorphous or glassy carbon),^75^ decreasing specific surface area,^29,39^ and by utilizing proper electrolyte additives.^97^

(3) There have been significant advancements with respect of optimizing binder^15,16^ and electrolyte chemistry for the specific case of silicon.^17^ While the vast majority of the investigations has focused on the design and realization of nanostructures, novel electrolyte chemistries can be highly effective at stabilizing the performance of silicon-containing anodes. This is an area which is prime for further innovation.

(4) Most likely, there is not going to be a single approach that can solve all the issues related to silicon for anodes. A combination of nanostructure design and synthesis, together with the development of specialized binders and electrolyte additives, will be necessary to achieve stability that meets commercial standard. The authors recommend stronger collaborations within the community. Teams that can integrate expertise in materials science, process engineering, electrochemistry and organic chemistry will have a higher probability of success.

While this field poses challenges from a fundamental science point of view, it is ultimately strongly application driven. Therefore any solution proposed by the scientific community will ultimately have to withstand the test of commercialization. For that reason, it is necessary to keep scalability issues under consideration early in the technology development stage. Any proposed solution will have to include scalable processing steps. The cost and quality of silicon powders are major issues that deserves further investigation (see Fig. 9 and 10), with this application space being very sensitive to size distribution effects. More efforts need to be devoted towards developing a nanoparticle production system that can achieve both a tight size distribution and is compatible with tons per year processing scale.

## Conflicts of interest

There are no conflicts to declare.

## Supplementary Material
